# Robot-assisted thoracoscopic resection of a posterior mediastinal tumor with preserving the artery of Adamkiewicz

**DOI:** 10.1186/s40792-022-01487-6

**Published:** 2022-07-06

**Authors:** Yukiko Nemoto, Koji Kuroda, Masataka Mori, Masatoshi Kanayama, Tiaji Kuwata, Masaru Takenaka, Fumihiro Tanaka

**Affiliations:** Second Department of Surgery (Chest Surgery), University of Occupational and Environmental Health, 1-1 Iseigaoka, Yahata-higashi-ku, Kitakyushu, 8078555 Japan

**Keywords:** Posterior mediastinal tumor, Robot-assisted surgery, RATS, Artery of Adamkiewicz

## Abstract

**Background:**

The artery of Adamkiewicz (AKA) provides the major blood supply to the lower two-thirds of the spinal cord. As the AKA typically arises from a left posterior intercostal artery at the levels between 9 and 12th thoracic vertebrae, injury of the AKA during thoracic surgery such as resection of a lower paravertebral tumor may cause serious neurological complications. Robot-assisted thoracic surgery (RATS) has several advantages over video-assisted thoracic surgery including three-dimensional and high-definition view with high image magnification and reduced restriction in movement of surgical instruments. Here, we present a case of a left paravertebral ganglioneuroma originating from the sympathetic trunk. Whereas both tumor-feeding arteries and the AKA arose from the 9^th^ intercostal artery, complete tumor resection with preserving the AKA was achieved by RATS.

**Case presentation:**

A 15-year-old girl admitted for surgery for a posterior mediastinal tumor. Chest computed tomography showed a well-circumscribed 8.0 cm tumor adjacent to 8–11th thoracic vertebrae and the descending aorta. Contrast-enhanced CT and angiography revealed that the AKA arose from the left 9^th^ intercostal artery that ran between the tumor and the vertebrae and that tumor-feeding arteries also arose from the same intercostal artery. RATS was performed with the left intercostal approach using the da Vinci Xi system (Intuitive Surgical, Mountain View, CA). The tumor originating from the sympathetic trunk was completely resected with preserving the sympathetic trunk and the AKA. Postoperative course was uneventful without any adverse event, such as neurological complications. The final pathological diagnosis of the tumor was ganglioneuroma.

**Conclusions:**

RATS is a useful surgical approach for removal of a mediastinal tumor with preserving surrounding organs or tissues, such as the AKA.

**Supplementary Information:**

The online version contains supplementary material available at 10.1186/s40792-022-01487-6.

## Background

The artery of Adamkiewicz (AKA), also known as the great anterior radiculomedullary artery, provides the major blood supply to the lower two-thirds of the spinal cord. As the AKA typically arises from a left posterior intercostal artery at the levels between the 9th and 12th thoracic vertebrae, injury of the AKA during thoracic surgery such as resection of a lower paravertebral tumor may cause serious neurological complications [1, 2].

Video-assisted thoracic surgery (VATS) has been widely employed to remove a benign mediastinal tumor. Robot-assisted thoracic surgery (RATS) has several advantages over VATS, which may provide easy access to the small mediastinal space, which enables safer and more precise removal of a mediastinal tumor surrounded by important organs and tissues [3–5].

Here, we present a case of a left paravertebral ganglioneuroma originating from the sympathetic trunk. Whereas both tumor-feeding arteries and the AKA arose from the 9th intercostal artery, complete tumor resection with preserving the AKA was achieved by RATS.

## Case presentation

**Fig. 1:**
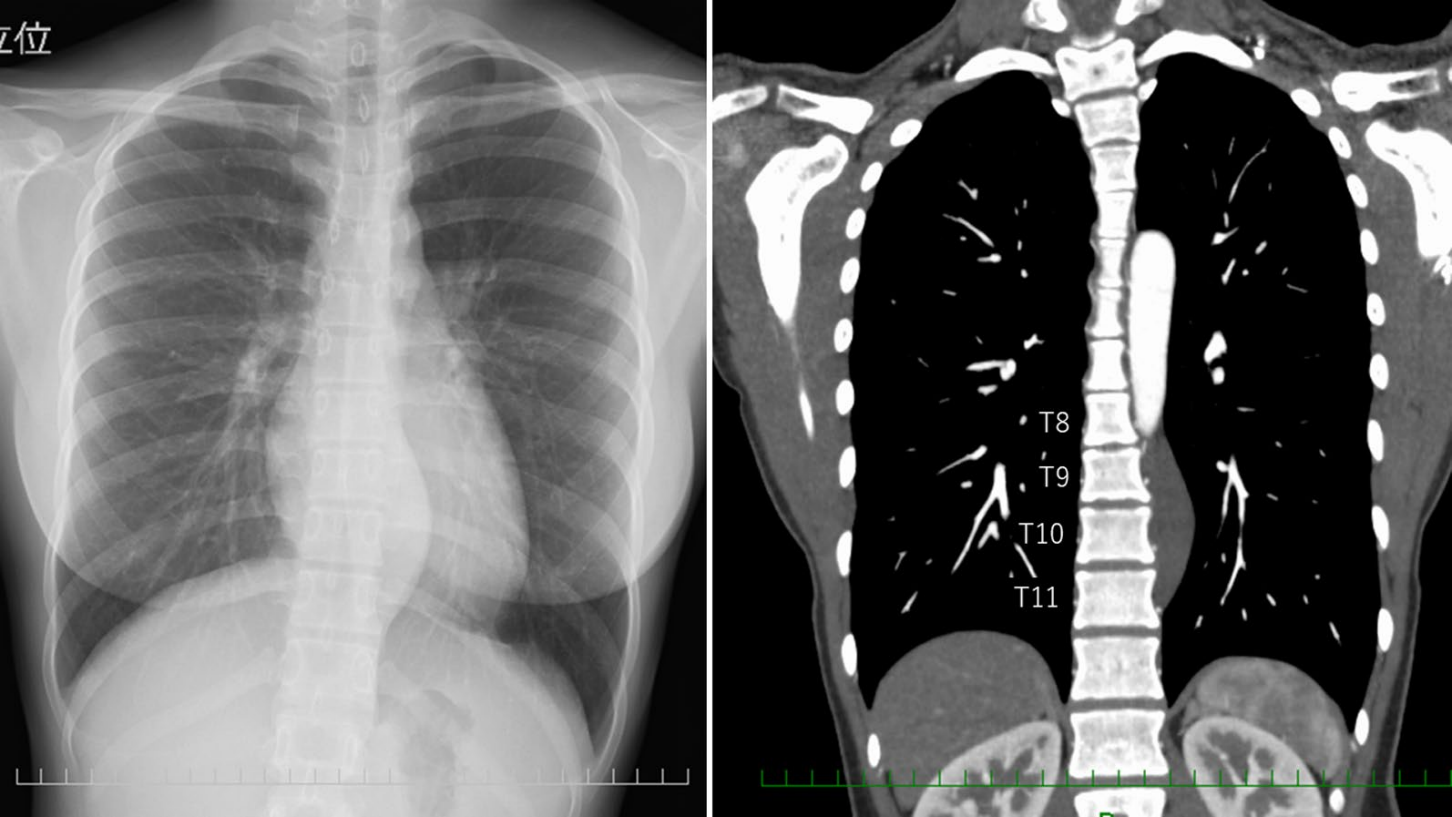
Chest roentgenogram (left) and computed tomography (right) showed a large paravertebral mass. T8, 8th thoracic vertebra; T9, 9th thoracic vertebra; T10, 10th thoracic vertebra; T11, 11th thoracic vertebra

**Fig. 2:**
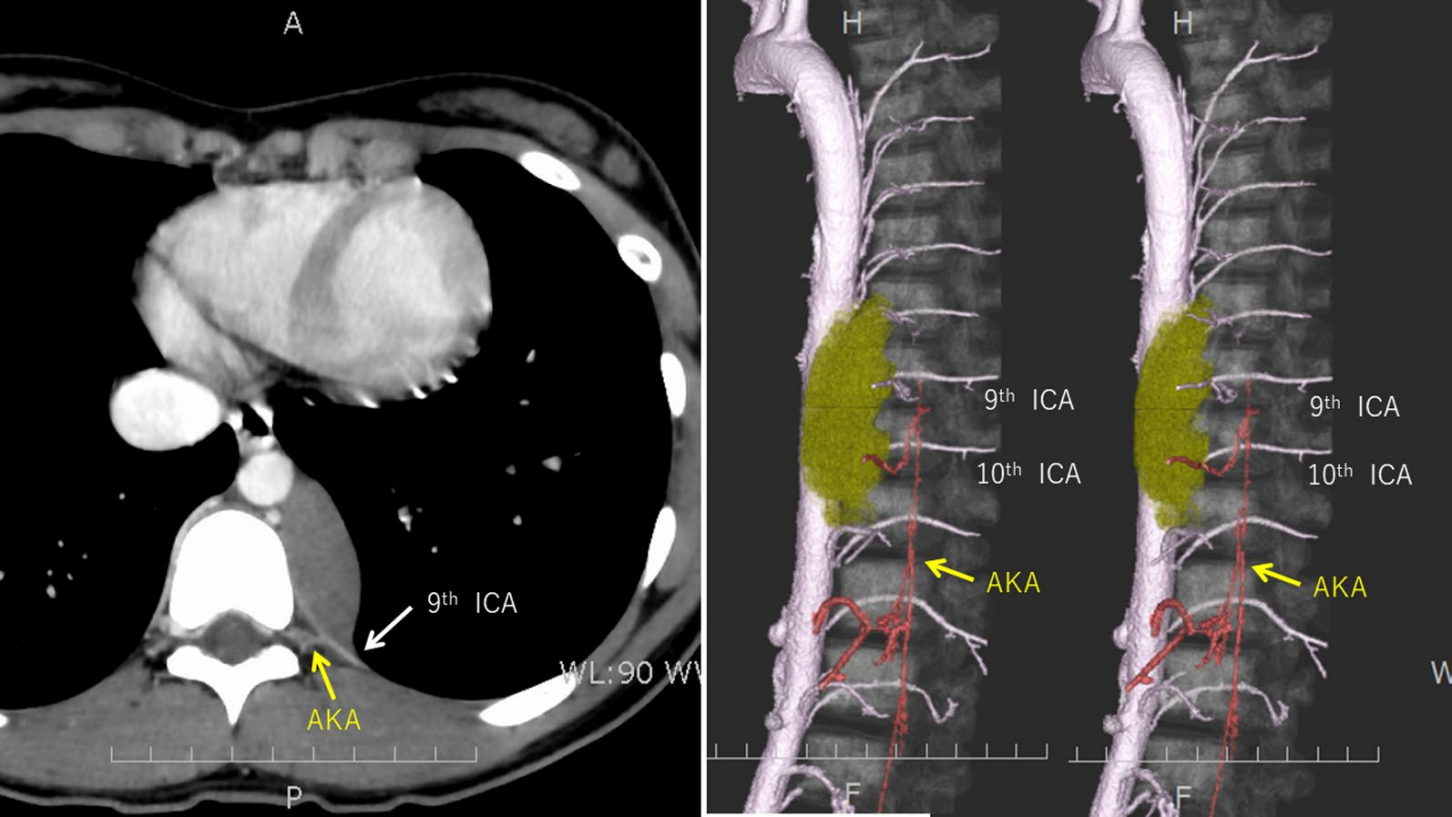
Contrast-enhanced computed tomography (CT) showed that the artery of Adamkiewicz arose from the 9th intercostal artery that ran between the tumor and the vertebrae. AKA, artery of Adamkiewicz; ICA, intercostal artery

**Fig. 3:**
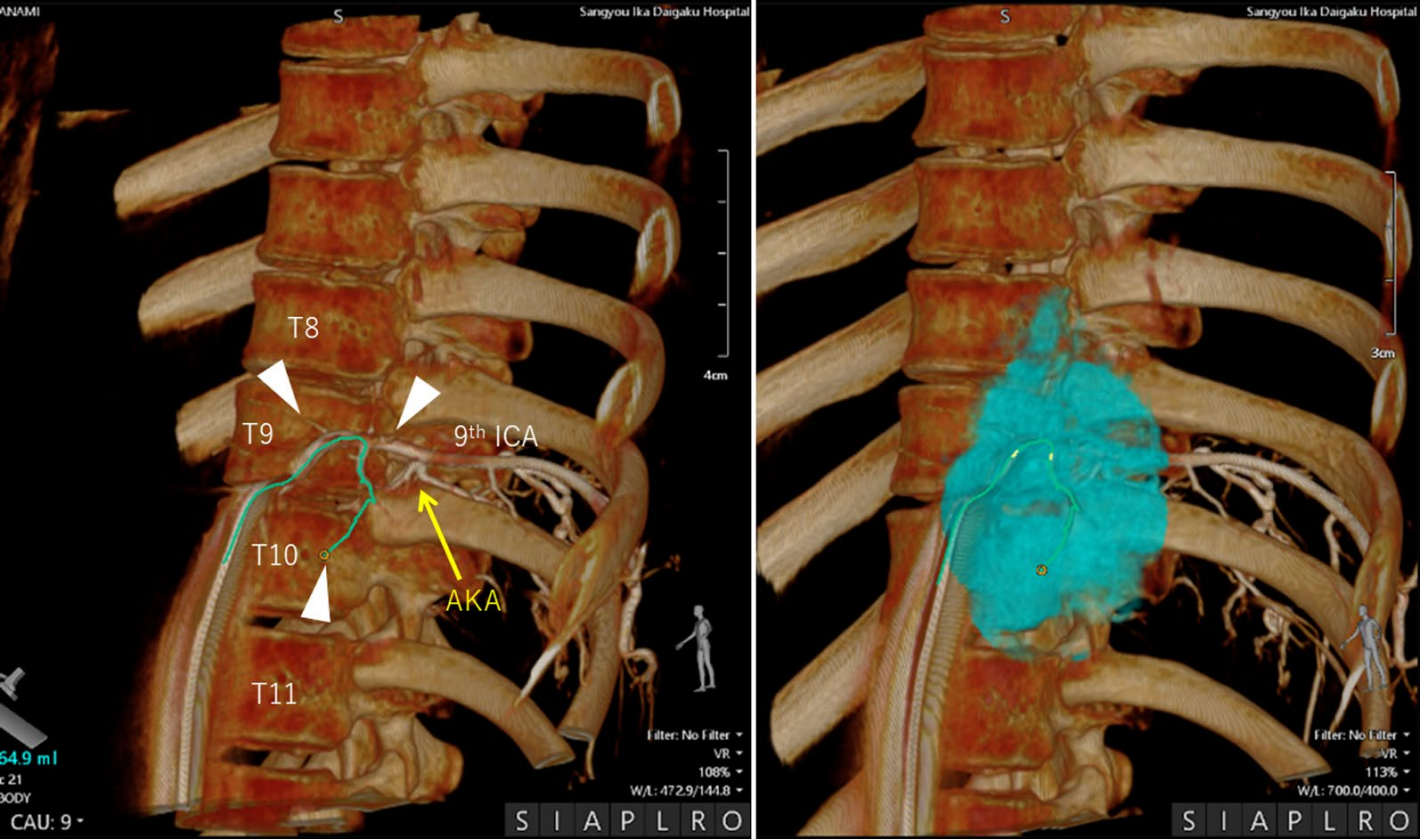
Digital subtraction angiography showed that tumor-feeding arteries (white arrows) and the artery of Adamkiewicz arose from the 9th intercostal artery. AKA, artery of Adamkiewicz; ICA, intercostal artery; T8, 8th thoracic vertebra; T9, 9th thoracic vertebra; T10, 10th thoracic vertebra; T11, 11th thoracic vertebra

A 15-year-old girl presented with back pain. A chest roentgenogram and computed tomography (CT) showed a well-circumscribed 8.0 cm tumor adjacent to the 8–11th thoracic vertebrae and the descending aorta in the left posterior mediastinum (Fig. 1). Contrast-enhanced CT revealed that the AKA arose from the left 9^th^ intercostal artery that ran between the tumor and the vertebrae (Fig. 2). Digital subtraction angiography (DSA) showed that tumor-feeding arteries also arose from the left 9^th^ intercostal artery (Fig. 3), which indicated that preservation of the 9th intercostal artery might be essential to avoid severe neurogenic complications, such as paraplegia.

The patient was placed in the right lateral decubitus position. RATS was performed with the left intercostal approach using the da Vinci Xi system (Intuitive Surgical, Mountain View, CA) with four arms. During the procedure, carbon dioxide gas was insufflated into the thoracic cavity through a trocar at the pressure of 8 mmHg. The camera trocar was first inserted at the 8th inter costal space (ICS) through middle axillary line. Then, a 3-cm utility thoracotomy was performed in the 6th ICS along the anterior axillary line. Two additional trocars were inserted as follows: one in the 9th ICS along the posterior axillary line and another in the 9th ICS along the subscapular line.

**Fig. 4:**
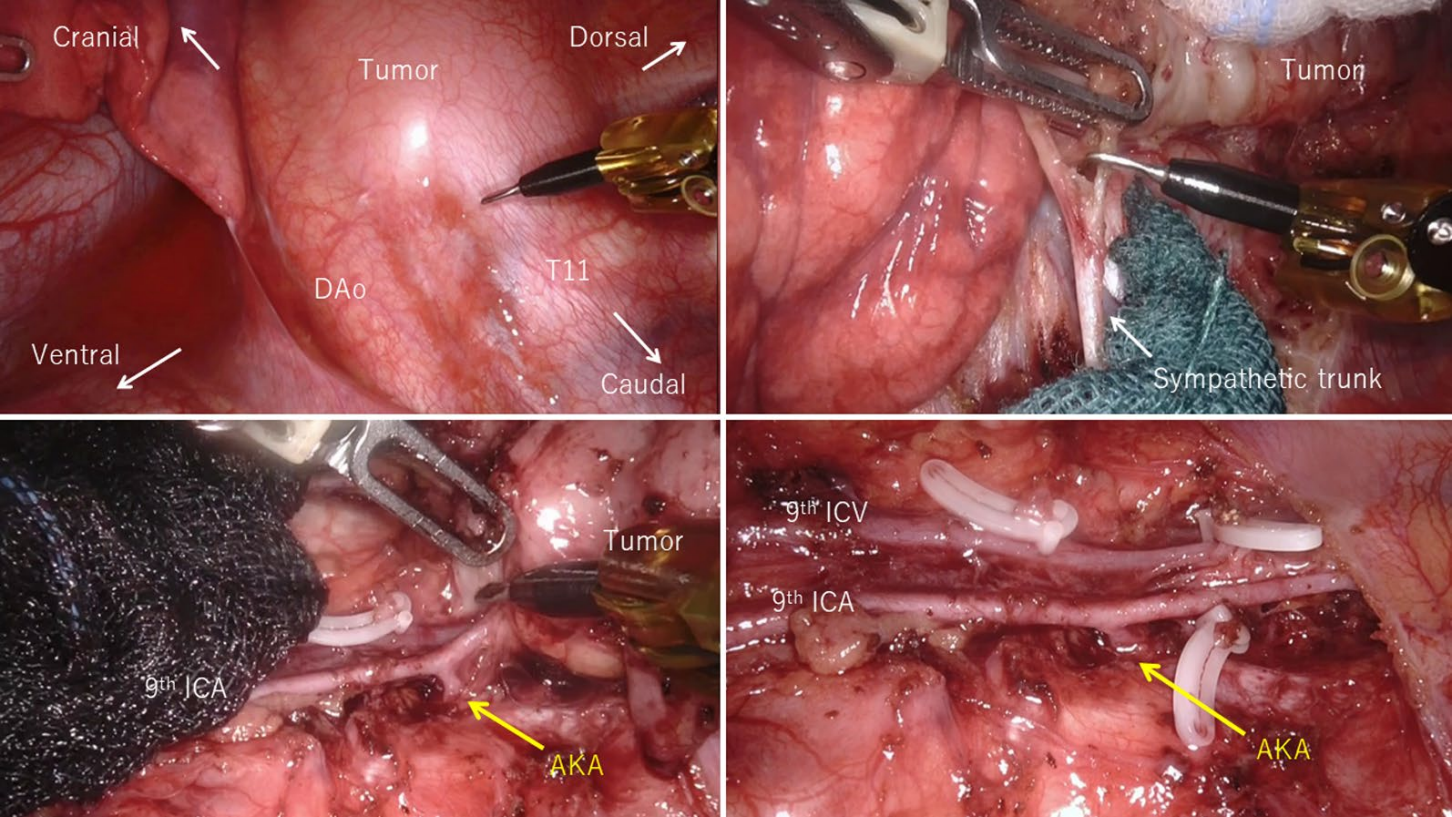
Well-circumscribed large paravertebral tumor, which originated from the sympathetic trunk, was identified (left upper and right upper). The nerve of origin was divided from the sympathetic trunk (right upper). Tumor-feeding arteries arose from the 9th intercostal artery (ICA) were dissected with preserving the artery of Adamkiewicz (AKA) (left lower). Complete tumor resection was achieved by robot-assisted thoracic surgery (RATS) with preserving the AKA as well as the 9th ICA and intercostal vein (ICV) (right lower). T11, 11th thoracic vertebra; *AKA* artery of Adamkiewicz, *ICA* intercostal artery, *ICV* intercostal vein

After the da Vinci system was docked, careful dissection was accomplished utilizing the Fenestrated bipolar forceps, the Tip-up fenestrated grasper and the Permanent cautery spatula (Intuitive Surgical) (Additional file 1: Video S1). The parietal pleura all the way around the tumor was first opened to mobilize the tumor, which revealed that the tumor originated from the sympathetic trunk. The nerve of origin was divided from the tumor with preserving the sympathetic trunk. The 9th intercostal vein as well as the 10th intercostal artery and vein were also preserved with dividing vessel branches involved by the tumor. The 9th intercostal artery was carefully dissected to avoid the injury of the AKA; tumor-feeding arteries arising from the 9th intercostal artery was clipped and divided, which achieved complete tumor resection with preservation of the AKA and the entire 9th intercostal artery (Fig. 4).

Postoperative course was uneventful, and back pain that she had complained of before surgery has been relived. The final pathological diagnosis of the tumor was ganglioneuroma. The patient is well without any complication at 18 months after surgery.

## Conclusions and discussion

We presented a case of a paravertebral neurogenic tumor which was successfully resected by RATS with preserving the AKA. This patient had complained of back pain probably associated with the tumor, which has been relieved after surgery.

Recently, RATS has been increasingly employed as a minimally invasive surgery for resection of a mediastinal tumor owing to several advantages over VATS. A single-institutional retrospective study of 130 patients with a posterior mediastinal neurogenic tumor resected by RATS (*n* = 58) or by VATS (*N* = 72) showed that RATS approach was significantly associated with a shorter intraoperative blood loss (85.8 mL versus 95.3 mL; *P* = 0.040) and a shorter postoperative hospital stay (2.2 days versus 2.4 days; *P* = 0.040). Concerning the incidence of postoperative complications, there was no significant difference between RATS approach and VATS approach (5.2 vs. 9.7%) [4]. A retrospective analysis of nation-wide database, which included 856 patients who underwent a minimally invasive surgery (RATS or VATS) for a mediastinal tumor in the United States, also showed a shorter hospital stay in the RATS group (3.8 days versus 4.3 days; *P* = 0.01). In addition, conversion to an open procedure was less frequent in the RATS group (4.9 vs. 14.7%; *P* < 0.001). Most importantly, the analysis revealed that RATS procedure was significantly associated with a lower incidence of adverse events (36.7 vs. 51.3%; *P* < 0.001); a multivariate analysis also showed that RATS procedure was independently associated with a decreased incidence of adverse events (odds ratio, 0.45; *P* = 0.001) [5].

These results clearly indicate the safety and feasibility of RATS in resection of a mediastinal tumor. In general, RATS may provide improved clinical outcomes, such as a lower incidence of postoperative adverse events. However, a specific advantage of RATS in preservation of the AKA to avoid neurological complications, which might be expected, has not been reported. A retrospective review reported that the incidence of paraplegia caused by injury of AKA during resection of mediastinal neurogenic tumor by open thoracotomy was 3.2% [6]. However, detailed data on AKA injury during mediastinal tumor resection by modern minimally invasive surgery are not available. Paraplegia may usually develop after resection of paravertebral tumor between the levels of the 5th thoracic vertebrae and the 1st lumber vertebrae [7, 8], but may develop after resection of apical paravertebral tumor [9]. Accordingly, careful preoperative evaluation and careful dissection to avoid AKA injury are essential in surgery for posterior mediastinal tumor regardless of the level of the tumor. In the present case, preoperative contrast-enhancement CT and DSA demonstrated that both the AKA and tumor-feeding arteries originated from the 9th intercostal artery. The tumor-feeding arteries were selectively and accurately divided by RATS with preserving the AKA as well as the 9^th^ intercostal artery and vein. As a result, the tumor was completely resected without any complication. Whereas a successful resection with preserving the AKA may be achieved by VATS, the present case suggest that RATS approach contributes to a safer minimally invasive resection of a paravertebral tumor with preserving the AKA.

To conclude, RATS is a useful surgical approach for removal of a posterior mediastinal tumor with preserving surrounding organs or tissues, such as the AKA.

## Supplementary Information

**Additional file 1.** Robot-assisted thoracoscopic resection of a posterior mediastinal tumor with preserving the artery of Adamkiewicz.

## Data Availability

The data sets supporting the conclusions of this article are included within the article and its additional files.

## Abbreviations

AKA: Artery of Adamkiewicz
VATS: Video-assisted thoracic surgery
RATS: Robot-assisted thoracic surgery
ICS: Intercostal space
